# Refugees’ Agency: On Resistance, Resilience, and Resources

**DOI:** 10.3390/ijerph19020806

**Published:** 2022-01-12

**Authors:** José Renkens, Els Rommes, Maria van den Muijsenbergh

**Affiliations:** 1 Department of Primary and Community Care, Radboud University Medical Centre, 6500 HB Nijmegen, The Netherlands; Maria.vandenMuijsenbergh@radboudumc.nl; 2 Gender & Diversity Studies, Radboud University, 6525 GD Nijmegen, The Netherlands; Els.Rommes@ru.nl; 3 School of Oganisation and Development, HAN University of Applied Sciences, 6525 EM Nijmegen, The Netherlands; 4 Pharos, Dutch Centre of Expertise on Health Disparities Utrecht, 3507 LH Utrecht, The Netherlands

**Keywords:** refugee decision-making agency, inclusiveness, responsiveness, equity, client- and person-centred health care, social inequalities

## Abstract

This study set out to answer the question ‘Which kinds of agency do refugees perform when dealing with mental health problems of themselves and their children?’. Aiming to gain more insight in why it seems harder for refugee parents and minors than for the native population to talk to health professionals about their mental health and wellbeing, we combined two theoretical notions of agency to investigate a broad spectrum of informants’ behaviour. We conducted 25 interviews with 30 refugees from 8 countries (Syria, Yemen, Iran, Afghanistan, Armenia, Eritrea, Turkish Kurdistan, Vietnam), whose Dutch residence permit varied from 26 years to less than one year. Data were analysed through open and axial coding, followed by pattern analyses. Although sometimes refugees seek (mental) healthcare, at other times they show agency by doing ‘nothing’ or by deliberately using distracting activities to deal with severe stress. Making use of resources available to them, oftentimes refugees show agency in ways that are less visible to healthcare professionals, by surviving, showing resilience, and suffering. In these cases, we think healthcare for refugees should intervene in a non-medical way, e.g., by supporting them to obtain resources that help refugees to (re)gain agency.

## 1. Introduction

Because of ongoing outbreaks of war, human rights violations, and persecution across the globe, more people than ever are forced to resettle in unfamiliar countries. Numerous studies have documented how refugees and their children are likely to have more, and more severe mental health problems (in this study, we use the terms mental health problems, mental distress, and psychosocial problems, interchangeably) because of experiences before, during, and after their flight [1,2,3,4,5,6,7,8]. They are known to be at risk of experiencing cumulative stress because of daily stressors and because of poor social and financial circumstances and migration-related problems [9,10].

For refugee parents and minors, it appears to be difficult to talk to healthcare professionals about their mental problems [3,11,12]. A recent study in the Netherlands among the general population shows several reasons for this: not being able to properly articulate problems and wishes; the feeling that the social or healthcare professional does not know you well; the professional’s opinion about a person’s capability to make a decision [9,13,14]. At the same time, healthcare professionals such as General Practitioners (GP) and mental health nurses indicate they have difficulties in recognising and discussing the mental distress of refugee children and adolescents [15,16,17,18,19,20,21]. Reasons refugees mention for not wanting to visit Dutch healthcare providers are cultural differences and lack of confidence in Dutch healthcare organisations [3,22,23]. As a result, refugees and their children are often under the radar of their GP when it comes to identifying, treating, and/or referring to mental healthcare [1,3,24]. Several studies have focused on how to improve access to and quality of (mental) healthcare for refugees in the Netherlands [12,25].

Working towards improvement of (mental) healthcare for refugees and their children, we see ourselves confronted with a lack of connection between psychological suffering on the one hand and the existing mental healthcare, on the other. In this study, we would like to contribute to health equality by providing insight in the ways in which refugee parents respond to their and their children’s mental distress, so that service providers can adjust their services to better fit refugees’ capacities and needs. To explore the different ways that refugee parents respond to mental distress, we will use the lens of agency, with which we mean ‘the capacity for purposive action, the ability to pursue goals, express voice and influence and make decisions’ [26]. This definition is based in a large body of feminist theorisation about agency, which expands more conventional ideas about agency as having power, privilege and emancipatory potential [27,28,29,30,31], to include agency of people that are not in a powerful position to improve personal circumstances, let alone those of the people of their community. Van Eerdewijk et al.’s definition is at the heart of their conceptual model for empowerment of women and girls, to work towards gender equality. Although refugees are a different group, they are too an underprivileged group that finds itself often in a disempowered situation. Empowerment is defined as a process as well as an outcome of the transformation of power relations by strengthening people’s choice and voice [26]. Besides individual ways to become empowered that are interesting for our study, the model also focuses on societal components for empowerment.

Therefore, we will not use the complete definition of Van Eerdewijk and adapt the model to make it useful for our analyses. In their model, they highlight three expressions of agency: decision-making, leadership, and collective action. In our case, where we explore refugees’ responses to their and their children’s mental problems, we do not expect extensive examples of leadership and collective action. We will explore our data for these as well, but we expect to find more expressions of decision-making as this is more fitting to individual problems. Furthermore, to assure we will include expressions of decision-making in the broadest sense, we will refine the model with another notion on agency, of Saba Mahmood, which we will explain further.

In the context of seeking and finding support or relief for mental distress, notions of agency would include being able to recognise mental distress, set individual ‘mental health goals’, and take action to realise these. Agency, therefore, traditionally seems to exist only as performance in the sense of observable behaviour [32], as a struggle [33]: this assumes that people not only set goals but also take action to pursue them [34]. For healthcare professionals, this would mean that agency could become visible in help-seeking behaviour or for instance in actively voicing rejection about offered help. Mahmood (2001) and Madhok et al. (2013) suggest, however, that agency is not always measurable or subversive: it consists of multiple actions and strategies that people use during challenging situations [29,33,34,35]. Sabah Mahmood, who draws on Judith Butler’s extensive writings about agency, takes the concept further: although Butler wants to steer clear of agency as ‘always and only opposed to power’ [36] (p. 17), Butler’s work still is mostly ‘derived from, and directed at articulations of resistance to social norms and the subordinating function of power’ [28] (p. 211). This did inspire Mahmood to explicitly extend the concept of agency beyond emancipatory ideals and she states: ‘what may appear to be a case of deplorable passivity and docility from a progressive point of view, may very well be a form of agency […] that aim[s] toward continuity, stasis, and stability’ (p. 212). Sarah Bracke calls this performance of agency ‘subaltern resilience’, ‘born out of the practice of getting up in the morning and making it through the day in conditions of often unbearable symbolic and material violence’ [37] (p. 60). In our case, agency, or (subaltern) resilience, refers to the multiple ways refugees use to deal with their mental distress. This means that we can consider refugees’ reactions to stress without looking for a predetermined goal or judging the ends of these responses. Instead, we can indeed regard them as they are: acts that seem appropriate to them in response to difficult or even hazardous times.

To refine Van Eerdewijk’s model of agency as our analysis tool, we include the view of Saba Mahmood on agency-as-resilience as multiple ways in which people engage with norms, including expressions of suffering and survival [28]. According to Van Eerdewijk et al., decision-making is an expression of agency through the ‘determined use of resources in and through decisions’ and ‘being able to act on them’. It also includes ‘voicing interests and concerns’ and ‘becomes visible in resistance, bargaining, negotiation, and reflection’. Combined with Mahmood’s notion of agency, we will look for those performances of agency that are more difficult to recognise as agency because they appear non-assertive and are more difficult to measure—if at all. Moreover, we are interested in explicating those ways of performing agency that remain invisible for healthcare professionals precisely because they are not directed at explicitly seeking help. In our analyses, we will thus look for instances of decision-making that are not necessarily translated into external-focused behaviour, but can be considered as strategies and actions that show or work towards resilience, or that people take in challenging situations. For example, ‘resistance’ can be voiced clearly but also be kept private, or seem withdrawn. Undergoing the experience of pain (grief, trauma) is not limited to passive suffering, but also means to actively endure hardness, contemplate, be courageous and practice patience, as part of being pious; ‘inhabiting ethical norms and moral principles’ as to survive within a system of inequality [29]. For religious people, ‘endurance’ can be understood as ‘bearing and living hardship’; in secular examples, it can be found in ‘seeking self-empowerment through the cultivation of self-esteem’ (for example by pursuing a professional career). Resilience, often described in mental healthcare as the ability to deal with adverse circumstances, encompasses decisions about personal behaviour.

As explained earlier, we do not expect to find the other two expressions of agency as mentioned by Van Eerdewijk et al. in our population, though we will leave the option open to encounter them during analyses. Leadership is expressed through actively taking initiative to improve one’s circumstances and those of others, reaching out beyond the family circle. Collective action is expressed through ‘organising and mobilising’, ‘gaining and using solidarity’, ‘taking action collectively’ to ‘improve circumstances and position of one’s social group’, and ‘coming together around common goals and interests’.

We view agency in its context: people are not equally equipped in their ability to take a strong position in decision-making or negotiation processes, due to their intersecting social markers such as class, race, gender, and other social divisions [30,31]. The context and social groups people belong to provide access to various *resources* [31]. Agency and resources mutually reinforce each other and shape the possibilities for changes in the lives of disempowered groups. A range of different material, human, and social resources can be used in order to be able to perform (various kinds of) agency [31]. Within decision-making, resources are defined as “tangible and intangible capital and sources of power” that people “have, own or use individually or collectively in the exercise of agency” [26]. The first is ‘Critical consciousness’: an explicit awareness and questioning of how power relations influence people’s lives and how they act upon this, showing and strengthening self-awareness, self-respect, and self-confidence. The second is ‘Bodily integrity’ which encompasses not just health as defined by the World Health Organisation in 1946, as “a state of complete physical, mental and social well-being and not merely the absence of disease or infirmity”, but also safety, security, and control over their bodies. It also encompasses aspects concerning nutrition and being well fed. The last resource is ‘Assets’: knowledge and skills; time; social capital; financial and productive assets; and also training and mentorship.

The use of resources is fundamental for decision-making. They interact and influence each other; they can be mutually dependent and reinforcing. For example, social capital in the form of strong social networks can contribute to social wellbeing, and the other way around. In our analyses we will look for the different sources of power that refugees mention when dealing with stress or working towards mental wellbeing.

Insight in refugee parents’ agency, and the resources on which their agency is based, can offer professional caregivers new opportunities to collaborate with refugees and their children and tailor their care to their needs, doing justice to their lived experience. With this study, we aim to answer the research question: Which kinds of agency do refugees perform when dealing with mental health problems of themselves and their children, and which resources do they use to perform this agency?

## 2. Materials and Methods

### 2.1. Study Design and Setting

This study is part of the larger ongoing “Empowerment” project aiming at the improvement of person-centred integrated primary care for refugee minors [38] and the affiliated participatory Shabab Akwa project in which refugees and other stakeholders co-create an intervention to empower the self-management of refugee parents regarding psychosocial problems in their children [39]. The research question of the underlying study demands individual, semi-structured interviews about ways of dealing with mental health issues. This allows us to explore topics derived from literature on the one hand and be open to new topics that may emerge during the interview on the other.

### 2.2. Study Population

Refugees were recruited through purposive sampling, using networks of the researchers and refugee doctors from Syria and Eritrea. We aimed for diversity in sex, educational level, country of origin, time since migration, and age of children at the time of arrival in the Netherlands. Data collection went on until theoretical data saturation was reached. Participants were compensated for offering their time with a EUR 15 gift card.

### 2.3. Data Collection

#### 2.3.1. Topic List

For both interviewers, we used a semi-structured topic list, based on the literature [7,16] and expert opinions from discussions with refugee doctors in our network. It included questions on experiences with recognition and handling of psychosocial problems and stress in their children and themselves, as well as their experiences with Dutch health care and social care professionals.

As the term ‘psychosocial problems’ could be unfamiliar and lead to misunderstanding, the term ’stress’ was used as well.

#### 2.3.2. Procedures

Both female interviewers have a refugee background themselves, and where possible, conducted the interviews in their mother tongue (Dari and Arabic); a few were in English and the rest in Dutch. They translated the non-Dutch interviews during transcription.

Interviews were conducted between September 2019 and March 2021, face-to-face or online due to COVID restrictions, and lasted between 45 and 90 min. All interviews were audio-recorded and transcribed verbatim and saved in Microsoft Word documents. Both interviewers checked the transcripts for accuracy.

### 2.4. Data Analyses

All transcripts were coded following an open and axial coding strategy [40] and analysed using pattern analyses through the lens of agency as explained in the Introduction. The first author, who led the pattern analyses, read all the interviews thoroughly and identified and marked those parts where participants discussed their own or their children’s psychosocial problems or stress. Additionally, ways of dealing with problems, and used or needed resources, were marked. The second author went through the interviews independently with similar questions, and together the first and second authors came to an agreement about which fragments might give us information about parents showing a certain kind of agency—or lack thereof. We copied all 126 selected fragments into a Microsoft Excel file and the first author summarised every fragment into one short sentence. These summaries were used to create a preliminary set of labels. We assigned one or more labels grounded in the content of the fragment (e.g., ‘hiking in nature’, or ‘finding distraction’) to every fragment. The next phase consisted of rereading and assigning codes to every fragment. The number of codes per fragment was unlimited; when necessary, a new label was created, and the earlier coded fragments were examined again. During the axial coding phase, patterns in the labels were sought and categorised. The labels were divided into three categories: the category WHAT, the category WHO, and the category HOW. In the WHAT category there are labels about *what subject* the participant talked about (such as work, shame, home), or *what action* they took (such as initiative, love and attention, seek help). In the WHO category, there are labels *about who* they are talking (such as self, child, partner, family), and *where* they find solace (such as religion, nature, physical exercise, school, health care professional). In the HOW category, labels for *emotions* or *experiences* were gathered (such as positive/optimistic, equal/self-confidence, angry, suspicious, rejection). This resulted in 17 labels in WHAT, 11 labels in WHO, and 14 labels in HOW. The second author checked all steps from the selection fragments to the labelling and categorizing of the labels.

**Figure 1:**
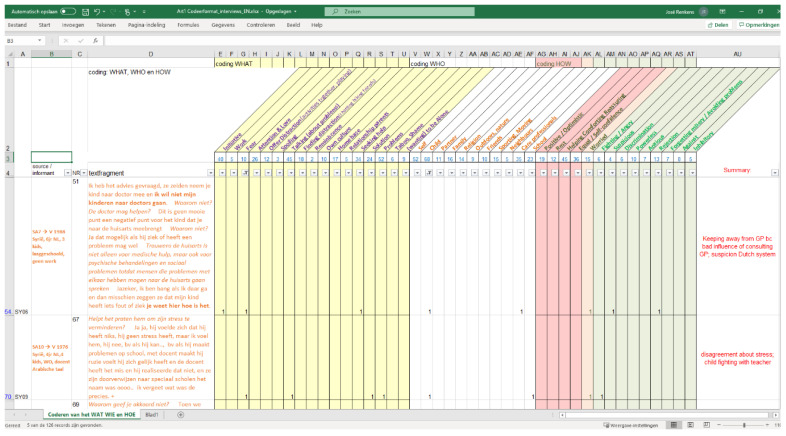
Excel screenshot with two filters applied (‘Fear’ and ‘Child’).

We used the filter function of Excel to make different combinations of labels and thus fragments, to enable the discovery of possible patterns in the narratives/answers of informants. Reading and rereading fragments overarching themes emerged, containing similarities and patterns in our participants’ answers. Please see Figure 1 for a screenshot of an example where two filters were applied (‘Fear’ and ‘Child’). Emerging patterns are examined in the light of agency, as explained in the introduction: distinguishing the different aspects of decision-making including answers that express endurance, suffering and surviving, and those that show or work towards resilience.

The findings are presented in a narrative format with the occasional interviewer’s question or remark written in italics.

### 2.5. Ethical Considerations

The study was approved by the Medical Ethics Committee of Radboud University Medical Centre in Nijmegen (file number 2019-5398).

Each interview was identified by a unique identifier code and all identifying information was removed from the transcripts.

As the subject was sensitive, specific attention was paid to avoid probing of questions and awareness of and respect for interviewees’ boundaries and possible emotional consequences. An independent general practitioner, experienced in caring for refugees with mental distress, was available in case interviewees felt the need to discuss their situation or wanted support after the interview.

As many interviews contain references to concrete actions, persons, and places, the transcripts are not available for open access.

## 3. Findings

### 3.1. Characteristics of the Study Population

In total, 25 interviews were included with 30 persons (4 sets of husband and wife, and 1 mother and daughter set): 21 women and 9 men from 8 different countries (see Table 1). The mean age for women was 40, and the mean age for men was 38, ranging from 20 up to 66 years. Respondents were predominantly highly educated: 19 persons. The time since when they possessed a Dutch residence permit varied from 26 years to less than one year. A total of 3 participants actually did not have children themselves, but were in close contact with refugee minors within the family.

We will discuss the results following the three expressions of agency: Decision-making, Leadership, and Collective action of Van Eerdewijk et al., combined with Mahmoods view on agency-as-resilience. We will conclude with a few examples in which people showed a lack of agency, or agency ‘under pressure’.

All fragments are accompanied by a code, e.g., [F26_SY04] in which the F stands for fragment-number (here, 26), plus the individual identifier code which can be found in Table 1.

### 3.2. Decision-Making

Regarding all fragments that fit the description of decision-making, two central themes in our informants’ behaviour emerged. Firstly, *distraction* (from problems/stress). People *find distraction* in nature, in listening to music and watching films, chatting with friends, actively evoking memories of the past, and taking part in physical activities. For their children, they *offer distraction* through playing games together, giving (extra) love and attention, spoiling them a bit, and finding nice activities outside the home.

The second theme is the opposite: *Focus* (focus on, or ‘staying with’ problems). People do this through praying, seeking seclusion, talking about problems with important others such as family or friends, talking with children about their problems, and finding professional help.

#### 3.2.1. Decision-Making: Finding and Offering Distraction

Some people relieve their stress by going outside and spend some time in nature, for example:

‘I like to walk between the trees.

The most important thing indeed is to leave the house,

the first 10 steps change my psyche completely.’

[F58_SY07]

‘Yes, walking in the woods and breathing fresh air,

that helps me to release stress and feel comfortable.’

[F26_SY04]

Both women perform agency through decision-making: they decide to go outside and are able to act on this decision. The resource they use here is *bodily integrity*: they have/use a body that is *healthy* enough to go for a walk without assistance, and they appear to feel *safe* enough to do so.

Other forms of distraction that show agency as decision-making are gaming, watching films, and listening to music. For example:

‘.. when I am too busy at work, or there are many things about my homeland

or something, then I go gaming, or sports, yes I do sports. Sports really help. (..).

Then, I do not think about it, then I concentrate on other things, sport helps, (…)

it makes me feel calm, and yes, happy, and with gaming too.’

[F87+88_SY11]

In addition to bodily integrity for sporting, this participant’s resources are *knowledge and skills*: because of his work as an IT person, he knows how to use computers and he has the skill to use gaming as a distraction to relieve his stress.

Another form of decision-making is distracting children when they are in a bad mood, by giving something nice to eat, allowing more freedom than usual, or ‘spoiling’ them a little bit. For this, people use the resource *reflection* (thinking of what would help the child to alleviate his bad mood) and *assets*, in the form of *financial capital* and *time*.

Some of our participants used their imagination for distraction, e.g., by actively making plans for the future together, or evoking nice memories from the past when they or their children are stressed, as we see in the following example:

‘We talk to them about interesting subjects,

let them remember the most beautiful days in Syria,

not the bad things that resulted from the war,

like their visits to their grandparents. So, we let them focus on the positive sides.’

[F28_SY02]

This participant as well as some others say they experience positive effects from sharing stories, from the past as well as from the future. Maybe we can also see the resource of *critical consciousness* through being aware of the importance to take control in the situation where a child feels stressed. Agency is then expressed through *reflection*, *voicing interests and concerns*, and *showing and working towards resilience*.

#### 3.2.2. Decision-Making: Focus

Whereas almost all participants mentioned they used some form of distraction when faced with stress, the opposite, focusing on their (sources of) distress, was less common. Several refugees we interviewed said they ’do not like to talk about themselves or their problems’. For parents with children, this seemed to be different. Almost all parents said they regularly take time to talk with their children about, e.g., their problems in school:

‘My daughter’s best friend was in a different group. So, I talked to her,

are you sad, or how do you feel when he goes to another group?’

F91_IR01

Focusing on the problem of their children rather than distracting them may also be a way in which to increase the resilience of their children, using *reflection*, and *knowledge and skills*.

There were several ways in which refugees would focus on their distress. Some informants said they use prayer to ease their minds, to *practice patience* and to restore trust in a positive future when they are in distress. For instance, this Syrian woman who combines prayer with physical movement:

‘When I pray, it is a mix of movement and recitation,

I read some syllables from Quran while I make moves, it is somehow like Yoga. I believe in what I read. In Quran are so many sermons, wisdom and lessons which relieve your heart and soul.

Then, you know deep in your heart that all the difficulties will also go away, God will be with you, help you, and the future will be better.’

[F27_SA5]

Some people actively seek solitude:

‘I will sit in my room alone for a while, when I feel discomfort I like to sit alone. I am sitting alone, with my discomfort.

I log out from whatsapp, I turn off the phone…

everything becomes irritating to my nerves.

I then need to be alone with myself in my room.

Then, I will read the Quran or pray, or do Tasspeeh [praise God]

Then, yes, I feel better.’

F70_SY09

Although ‘reading the Quran’, ‘making moves’, and ‘doing Tasspeeh’ can be seen as a way of distraction, the way these participants spoke about it is about focusing their attention inward, reflecting and obtaining wisdom from the Quran, and gaining trust in the future, both showing and working towards resilience.

Some of our respondents actively sought advice from family members or friends when they experienced mental distress, using *social capital* as a resource. They mentioned that others can give a different perspective on things, or that they can help because they have more (life) experience. Besides finding help in informal contacts, several respondents said they prefer consultation with health care professionals. Some people argue that if you take your problems seriously, you should not go to laymen; others mention the taboo surrounding psychological problems and their shame in speaking about their problems with people they know, and the benefit of anonymity with a professional.

Sometimes a professional seeks contact with a refugee family with a more or less forced offer for help, and the refugee must deal with this. Several felt that they had not been consulted or involved in the problem definition, or felt the solution does not fit with their beliefs. Similarly to this, we see a woman from Syria, a mother of four children of whom one, according to school professionals, showed ‘problematic’ behaviour. The school wanted to send the son to special education, which mother refused:

‘They never asked our opinion; they find it normal

and were surprised when I refused—

‘on the contrary, it would be better for my child, would be good for his morals’ [they said].

We are people who think this is shameful, my child cannot go to such a centre. It is shameful, not suitable for us.

They may have different insights, they may be right, but we do not accept it.

We have a different culture; the Arab culture exists inside us. We cannot integrate.’

[F74_SY09]

Here, we see decision-making through *resistance*: she refuses to consent to the transfer of her son.

We also found examples of people who explicitly do not seek professional help, because they perceive Dutch caregivers to not be emphatic and understanding enough about their situation, or because of cultural differences. An Armenian woman told us that parents from her country ‘will not consult the GP when their children experience psychological or social problems’:

‘Because the mentality is not very similar, unless the caregiver

is a foreigner too—or they think that the person, despite being Dutch,

sympathizes with the situation,

only then they will allow it.’

F104_AR01

In the case of mental distress in children, some respondents indicated they were afraid of seeking professional help, as they feared then their children would be labelled as ill:

‘but I do not want my children to see doctors.

I am afraid that if I go there, maybe they say

there’s something wrong with my child, or he’s sick.

You know how it is here.’

F51_SY06

This is a different illustration of an *action-not-to-do-something*, showing agency as *resistance*, using *critical consciousness* as resource.

We also found several examples of *reflection* on how people from different cultures experience taboos on talking about psychological problems, also in the younger generations of refugees. One of our respondents indicated that he looks for a balance between his home culture and the culture of the host country: a ‘middle ground’. He saw complete integration as undesirable because it may lead to struggles in communication with older community members. Finding a ‘middle road’ and switching back and forth is an example of decision-making through *bargaining*, using *knowledge and skills* as resources.

### 3.3. Leadership and Collective Action

Although the interviews aimed at gathering individual responses to mental distress, we did find one person who showed agency through leadership: a Syrian woman who founded an organisation to help and educate fellow refugees about mental health problems in their children. She made use of *assets* as resource, in the form of *social capital* and *knowledge and skills*.

‘Actually, I have founded an organisation,

that can take care of mental health problems in refugee children

about which the parents need more education.

I need professionals to give courses about these issues to refugees.

Through my organization I can provide the public with more education

and support for refugees parents.’

F31_SY04

*Showing initiative to improve circumstances of people beyond her family circle*, is an example of leadership. In this way, she provides resources for other parents: they can work towards resilience by making use of her organisation.

The same woman who served as the one example of leadership, we can use as the only example for collective action: she mobilises professionals and tries to create a link between professionals and refugee parents. To do so, she uses several resources such as *knowledge and skills*, *social capital* (*network*), and *critical consciousness*:

‘We are an organisation that can give this information

but it needs to reach the public of refugees, there is a missing link in the circle between [refugees and professionals]. So we can offer the connection

between the group of refugees and the professionals.

We know more about the problems of refugees, and

Dutch organizations know more about solutions,

so, the refugee organizations must be activated.

We as newcomers can do primarily a lot for refugees and this society regarding refugee issues.’

F32a_SY04

### 3.4. Agency under Pressure/Suffering & Surviving

In our interviews, respondents also showed examples where they seemed to lack agency. Sometimes they explicitly stated that they do not know where to go for help, or they do not know what kind of help is available: they lack resources such as *knowledge* or *social capital*. Sometimes people go through such difficult times that they are unable to seek help, like a woman whose husband suffered from severe depression, and he did not talk to their daughters and son anymore. The mother was in survival mode, and she said she could not organize activities to help her children because she was so worried about her husband: she could never let him out of sight. Through our lens of agency, we see a lack of resources that would help in their current situation. In this example, they could have benefitted from *bodily integrity*: *mental and social wellbeing*, *safety*, and *security*.

Some respondents felt powerless about their situation and stopped performing agency. This ‘giving up’ fits in the notion of *suffering* as Mahmood describes, which would make it a (small) performance of agency.

‘We tried several things in this country but found no solution.

In this country, when a teacher writes something down

in his report about a child, there’s nothing you can do

There is no solution,

I felt injustice against my child (..)

Finally, I gave up.’

F69_SY09

Apparently, she felt she was out of resources to change her situation for the better: e.g., *bargaining power*. Finally, she gave up.

## 4. Discussion

### 4.1. Main Findings

To our knowledge, this is the first study on agency in refugees dealing with mental health problems of themselves or their children. Of the three expressions of agency, we found a broad range of examples of decision-making in the form of distraction or focus. *Distraction* was about ‘going away’ from the problem, whereas *Focus* was about ‘staying with the trouble’. We found also examples of agency-as-resilience, related to suffering and surviving. Agency-as-resistance became clear in refugees explicitly refusing professional care, and we saw examples of action-not-to-do something, besides a few examples of lack of agency, or agency under pressure. Unexpectedly, we found an instance of leadership and collective action in our dataset.

### 4.2. Reflection on Agency and Comparison with Other Studies

As Mahmood [29] states, capacities such as endurance, survival, suffering, and persistence are often considered to be the opposite of agency, but can be seen as agency-as-resilience (p. 167). These are ways of showing agency as not-external-focused behaviour. In our findings, distraction as well as focus can help refugees to become or stay resilient. As a result, the agency of refugees may become invisible for professional caregivers: refugees may seem passive because their behaviours are not directed at consulting healthcare professionals. For healthcare professionals, cautiousness is called for, to prevent seeing or using agency solely when becoming manifest as *action* as the opposite of survival, suffering, and showing resilience. The fact that some informants decide not to ask or accept Dutch (mental) healthcare can also be seen as agency-as-resistance, resistance to the dominant Dutch concept of professional healthcare as the solution for all problems. For Dutch professional caregivers, it might be difficult to grasp that this is a form of active self-management, a deliberate choice.

Care professionals in the Netherlands are trained to stimulate the patient in self-management and aim at shared decision-making [41]. However, healthcare professionals often have specific ideas about what constitutes ‘good’ health behaviour. Healthcare professionals acting on these notions take a behaviour-change approach, which can result in overly paternalistic attitudes towards patients. Not cohering to professionals’ recommended behaviour can lead to (harm through) victim blaming and stigmatisation, even when people do not have (control over) the necessary resources to change. Although an empowerment approach is meant to work from the patient’s agency, some studies point out that it can still be a paternalistic tool for behavioural change, since its (imposed) goal is to empower people without consulting their needs or because of bias from the professional about a refugee’s ability for autonomy [42,43]. When empowerment is being ‘given’ by healthcare or social professionals instead of taken, true self-management—based on patients’ preferences and available resources—is jeopardised. We agree with Halvorsen et al., there is a need for turning the tables in who will take the initiative in self-management. Health professionals’ lack of awareness about different kinds of agency “may lead to a judgmental attitude and is a crucial barrier to empowerment” [43]. In case of mental distress, we could argue that ‘not wanting to talk about oneself’ is repeating the well-known trope that refugees do not talk about psychosocial problems, and that ‘western’ people are better suited to solve problems because they do talk.

In this article, we contribute to the work of feminist authors such as Ahmed and Bracke, who challenge the conception of agency as either resisting or upholding societal norms. Instead, our study makes room for ‘prosaic’ performances of agency by people in less powerful positions. It shows how refugees are navigating their lives within the given circumstances, in the best possible way, and how they often lack the needed resources to change norms and make a difference in their structural circumstances [44]. Similar to Paudyal et al. who found how some Syrian refugees became ‘their own doctor’, in turning to faith, rituals, and nature for healing and comfort [45], we found the agency of refugees did make a difference in their *personal* circumstances.

The fact that we did not find many examples of leadership and collective action in refugees, can be (partly) explained by the nature of the research topic: psychosocial problems in Dutch society are mainly seen as individual problems instead of collective problems. Moreover, we see refugees’ often insecure and powerless position in society caused, i.e., by a lack of (high quality) resources, as responsible for the absence of instances of leadership and collective action [46]. To improve refugees’ circumstances, it would be helpful to obtain the necessary resources they need/use to express decision-making, leadership, and collective action, to prevent mental health problems (as much as possible) through societal change at a larger scale. However, refugees themselves have little influence over how to obtain the resources necessary in this process. According to Teunissen et al. [12] migrants often attribute mental health problems to their poor living circumstances: “a normal reaction to abnormal circumstances”. Or, to speak with Butler [47]: as long as there are no substantial changes in the circumstances of ‘precarity’ that refugees themselves have no power over to influence, refugees as a ‘vulnerable group’ will have great difficulty in climbing out of an underdog position.

The one example we found in our data for leadership and for collective action aims at facilitating refugees to adapt better to the Dutch mental healthcare system, showing resilience in recovering or ‘fixing’ youth in order to fit into Dutch society. We are critical of the neoliberal explanation of ‘resilience’ as being able to ‘bounce back’ after emotionally intense or even traumatic events. It overpathologizes and denies the reality of heart-brokenness of many refugees; they are expected to behave ‘in such a way that does not cause too much inconvenience or trouble to their surroundings’ [48]. Barber et al. (2016) identified two cultural syndromes in Palestinians, of “feeling broken” and “feeling destroyed”. Despite these feelings, the persistent shared experience, determination, and resistance make the biomedical construct of “mental illness” an inappropriate label for the communal experience of traumatic political violence [49]. We argue with Sara Ahmed: when strengthening resilience means becoming stronger so one can bear more, not succumbing to pressure, even when this pressure keeps increasing, such that this pressure can indeed be gradually increased [50], then maybe we should not strive for resilience. Sarah Bracke takes a similar stance, in explaining that without the continuous presence of disaster—or at least a threat of it—there would be no need to insist on resilience [37] (p. 58–59). Instead, refugees need and deserve a strong agency, to take as much control as they want to (re)form their and their children’s lives, not necessarily in the way or direction that care professionals see fit.

### 4.3. Strengths and Limitations

The strengths of this study include the background of our interviewers: they both have refugee experience themselves which might have helped interviewees to open up about their experiences. However, possible bias could have arisen for the same reason: because the interviewees knew they were sharing a refugee experience with the interviewer, some things may not have been said, e.g., because they expected these to be already known or understood.

Applying the combined theories of agency based in feminist studies, to the experiences of refugees shows additional, positive ways in dealing with mental distress, even without the needed resources to make a difference, and sometimes different from healthcare workers’ expectations. To our knowledge, this is very novel in the field of refugee medicine and these insights provide novel constructive ways to deal with refugees for professional caregivers.

This study has limitations that are inherent to a qualitative approach, e.g., other authors might interpret data differently. We addressed this limitation through reflexivity and through analysing data individually first before discussing our findings. Another limitation is that two-thirds of our group of respondents consisted of people who had a university degree or a higher vocational education, which may have contributed to them having more reflexive skills. Additionally, recruiting people through our (predominantly medical) networks might have led to additional biases in the respondent group. This may have skewed the data because most respondents are somehow connected to at least one person with a medical background; they have the resource network/social capital. If these biases in our group of respondents will have influenced the answers, we expect our respondents to generally have been more positive and knowledgeable about professional care and possibly to use more externally focused kinds of agency.

### 4.4. Implications/Lessons Learned

Our understanding of agency, in particular with regard to the decision to not seek professional help as a form of self-management, can be beneficial for health and social professionals. They could use the insights about distraction and focus in their conversations with refugees and offer them examples that others have experienced to be helpful. A specific example can be distraction as a way to deal with distress in the form of recalling memories: telling (life) stories is already used in so-called narrative therapy [51] and it has been shown that these stories can help in (re) gaining self-confidence and reconstituting one’s identity. Other examples that became clear in our data are strengthening/improving refugees’ resources that are within the scope of health and social professionals, such as helping with social capital; offer housing that is close to people that are/can be part of their community; housing close to nature and with enough room to be alone when needed.

A person-centred approach would help to view refugees as persons with agency, even when this agency seems less strong or decisive. Taking their context and lived experience into account might open up different ways of offering help [22,52].

## 5. Conclusions

When dealing with mental distress, refugees perform agency in different ways. Some of our respondents explained they do not want to visit Dutch healthcare providers because of cultural differences and lack of confidence in Dutch healthcare organisation, which we also saw in earlier studies [3,22,23]. Besides sometimes seeking (mental) healthcare, they show agency by doing ‘nothing’—they find distraction and deliberately use activities that help them deal with stress. For this, they make use of the resources that are available to them under the current circumstances. They show agency in ways that are less visible to healthcare professionals, by surviving, showing resilience, and suffering. Insights in these kinds of agency may inform practices and policies that improve the quality of healthcare for refugees, for instance by intervening in a non-medical way, or by helping create or obtain resources that help refugees to (re)gain agency.

Future studies can provide more insight in how subgroups of refugees with, e.g., different levels of education and lengths of stay, may or may not perform different kinds of agency and have or have no access to (high quality) resources when dealing with mental distress. Larger scale studies can give insight into experiences of refugees with healthcare professionals who perform ‘care as usual’ compared with experiences with healthcare professionals who were specifically trained in the concepts of agency and the experiences of refugees. Action-oriented, participatory research can help us to gain insight into how to make high-quality resources more available, focusing on all three expressions of agency, not just in the everyday sense as we saw with ‘decision-making’. Strengthening agency in making strategic choices in life, as well as forms of agency that seek to question, challenge, and ultimately transform the status quo, should be included [31]. This would lead to refugees performing agency in terms of ‘leadership’ and ‘collective action’, improving lives beyond their family circle, in the way they themselves see fit in their context.

## Figures and Tables

**Table 1:** Overview of participants.

ID Code	Home Country	Male (M)/Female (F) + Age	Educational Level	Duration of Stay in NL * [in Years]	Number of Children *
SY01	Syria	F57	Lower vocational education	5	3
SY02	Syria	F36	University education	5	2
YE01	Yemen	F29	Lower vocational education	10	5
SY03	Syria	M33	University education	6	0
SY04	Syria	F41	University education	6	2
SY05	Syria	F39	University education	5	2
SY06	Syria	F32	Lower vocational education	6	3
SY07	Syria	F48	No education	5	4
SY08	Syria	M44	University education	6	2
SY09	Syria	F44	University education	4	4
SY10	Syria	F54	Higher vocational education	5	3
YE02	Yemen	F37	Higher vocational education	2	3
SY11	Syria	M40	Higher vocational education	6	3
IR01	Iran	F29	No education	5	1
AF01	Afghanistan	F42	Secondary school	12	4
AF02	Afghanistan	M31	University education	12	0
AF03	Afghanistan	F56	Higher vocational education	16	4
AR01	Armenia	F20	Vocational education	<1	0 **
AR02	Armenia (mother + daughter)	F66 F33	Vocational education University education	6 4	2 0
ER01	Eritrea (couple)	F33 M36	Higher vocational education University education	4 6	3
ER02	Eritrea (couple)	F34 M51	Secondary school Higher vocational education	3 4	4
ER03	Eritrea (couple)	F25 M29	Secondary school Secondary school	5 both	2
IR02	Iran	M49	Higher vocational education	9	1
KT01	Turkish Kurdistan (couple)	F31 M33	University education University education	13 both	1 (new-born)
VIE01	Vietnam	F55	Higher vocational education	16	3

* at time of interview; ** taking care of baby brother.

## Data Availability

Original data are available for other researchers through written request only, to ensure privacy protection of respondents.
